# Flea-borne rickettsioses: ecologic considerations.

**DOI:** 10.3201/eid0303.970308

**Published:** 1997

**Authors:** A F Azad, S Radulovic, J A Higgins, B H Noden, J M Troyer

**Affiliations:** Department of Microbiology and Immunobiology, University of Maryland School of Medicine, Baltimore, MD 21201, USA. aazad@umabnet.ab.umd.edu

## Abstract

Ecologic and economic factors, as well as changes in human behavior, have resulted in the emergence of new and the reemergence of existing but forgotten infectious diseases during the past 20 years. Flea-borne disease organisms (e.g., Yersinia pestis, Rickettsia typhi, R. felis, and Bartonella henselae) are widely distributed throughout the world in endemic-disease foci, where components of the enzootic cycle are present. However, flea-borne diseases could reemerge in epidemic form because of changes in vector-host ecology due to environmental and human behavior modification. The changing ecology of murine typhus in southern California and Texas over the past 30 years is a good example of urban and suburban expansion affecting infectious disease outbreaks. In these areas, the classic rat-flea-rat cycle of R. typhi has been replaced by a peridomestic animal cycle involving, e.g., free-ranging cats, dogs, and opossums and their fleas. In addition to the vector-host components of the murine typhus cycle, we have uncovered a second typhuslike rickettsia, R. felis. This agent was identified from the blood of a hospitalized febrile patient and from opossums and their fleas. We reviewed the ecology of R. typhi and R. felis and present recent data relevant to the vector biology, immunology, and molecular characterization and phylogeny of flea-borne rickettsioses.


### Flea-borne Rickettsioses: Ecologic Considerations

**Abdu F. Azad, Suzana Radulovic, James A. Higgins, B. H. Noden, and Jill M. Troyer**
University of Maryland School of Medicine, Baltimore, Maryland, USA

Ecologic and economic factors, as well as changes in human behavior, have resulted in the emergence of new and the reemergence of existing but forgotten infectious diseases during the past 20 years. Flea-borne disease organisms (e.g., *Yersinia pestis, Rickettsia typhi, R. felis,* and *Bartonella henselae*) are widely distributed throughout the world in endemic-disease foci, where components of the enzootic cycle are present. However, flea-borne diseases could reemerge in epidemic form because of changes in vector-host ecology due to environmental and human behavior modification. The changing ecology of murine typhus in southern California and Texas over the past 30 years is a good example of urban and suburban expansion affecting infectious disease outbreaks. In these areas, the classic rat-flea-rat cycle of *R. typhi* has been replaced by a peridomestic animal cycle involving, e.g., free-ranging cats, dogs, and opossums and their fleas. In addition to the vector-host components of the murine typhus cycle, we have uncovered a second typhuslike rickettsia, *R. felis*. This agent was identified from the blood of a hospitalized febrile patient and from opossums and their fleas. We reviewed the ecology of *R. typhi* and *R. felis* and present recent data relevant to the vector biology, immunology, and molecular characterization and phylogeny of flea-borne rickettsioses.

A complex matrix of ecologic and economic factors and human behavior changes has resulted in the emergence of new infectious diseases during the past 20 years. Ecologic changes lead to the emergence of both known and as yet unknown pathogens circulating in the complex host and vector systems of undisturbed habitats. A drastic increase of Rocky Mountain spotted fever in the late 1970s, Lyme disease in the early 1980s, and ehrlichioses in the 1990s in the United States attests to the strong correlation of these diseases to human-made ecologic changes and further illustrates the inability of existing monitoring systems to predict outbreaks and protect at-risk populations. Unmanaged growth and expansion of the suburbs into undisturbed habitats have generated ideal ecosystems for many displaced animals. The changing ecology of murine typhus in southern California and Texas over the past 30 years is a good example of suburban expansion and environmental modifications affecting infectious disease outbreaks. In suburban areas, vector fleas are most often associated with human habitation through their natural hosts, e.g., commensal rodents and peridomestic animals, such as free-ranging cats and dogs, opossums, raccoons, and squirrels. Fleas commonly found on these animals are picked up by household pets and brought into homes. These fleas, apart from being a nuisance, may carry pathogenic organisms of concern to human health.

Of the 2,000 species and subspecies of fleas, only a handful serve as vectors of human diseases. Several bacterial pathogens of public health importance are maintained and transmitted by fleas, among them, *Yersinia pestis*, the causative agent of plague (known in history as black death). Flea-borne human pathogens are maintained in a zoonotic cycle involving mammalian hosts and fleas. They seldom cause overt disease in their natural hosts but commonly result in clinical disease, e.g., plague, murine typhus, and cat-scratch disease, in humans. The rapid spread of pathogens to human populations is due to the frequent feeding behavior and

Address for correspondence: Abdu F. Azad, Department of Microbiology and Immunology, University of Maryland School of Medicine, 655 W. Baltimore Street, Baltimore, MD 21201 USA; fax: 410-706-0282; e-mail: aazad@umabnet.ab.umd.edu.

extraordinary mobility of fleas. Flea-borne diseases are widely distributed throughout the world, in the form of endemic foci, where components of the cycle are present; however, these diseases become epidemic in human populations when infected mammalian hosts die and their fleas leave in search of a bloodmeal. Of flea-borne bacterial pathogens (e.g., *Y. pestis, Rickettsia typhi, R. felis,* and *Bartonella henselae*), the most studied and reviewed is *Y. pestis*. In this article, however, we want to examine the agents of flea-borne rickettsioses, namely *R. typhi* and the newly identified *R. felis*. Beginning with *R. typhi* ecology, we will review and discuss recent data relevant to the ecology, vector biology, immunologic and molecular characterization, and phylogeny of the flea-borne rickettsiae.

### Natural History of Flea-borne Rickettsioses

"Murine typhus is a good example of a disease whose importance is not adequately appreciated—except by the patient, and, even today, in most parts of the world, he will never know what ails him because the diagnosis will not be made" (1). Murine typhus is one of the most widely distributed arthropod-borne infections endemic in many coastal areas and ports throughout the world. It occurs in epidemics or with high prevalence; is often unrecognized and substantially underreported; and, although it can be clinically mild, it can also cause severe illness and death (2,3). The severity of murine typhus infection has been associated with old age, delayed diagnosis, hepatic and renal dysfunction, central nervous system abnormalities, and pulmonary compromise. Death occurs in up to 4% of hospitalized patients (3). Thousands of human cases were reported annually in the United States (1,2). Outbreaks have been reported in Australia and recently in China, Greece, Israel, Kuwait, and Thailand (1,2,4). Recent serosurveys have demonstrated high prevalence of anti-typhus group (TG) rickettsiae in Asia and southern Europe (4). Despite the current level of reported human cases in the United States of fewer than 100 per year, murine typhus has been the subject of several recent studies (3,4). Rekindled interest in this disease is partially a result of field data and epidemiologic surveys, which have prompted a reconsideration of established components of the vector-reservoir cycle and their interaction with humans (5-7). The classic cycle of *R. typhi*, the etiologic agent of murine typhus, involves rats (*Rattus rattus* and *R. norvegicus*) and the rat flea, *Xensopsylla cheopis* (Figure 1). The flea has been considered the main vector, and the disease is transmitted by flea bites or contact with rickettsia-containing feces and tissues during or after blood feeding. Although the rat-flea-rat cycle is still the major route of human infection throughout the world, murine typhus exists in some endemic-disease foci where both rats and rat fleas are absent.

Reported cases of murine typhus in the United States are focused largely in central and southcentral Texas and the Los Angeles and Orange Counties in California; however, infected rats and their fleas are hard to document in these areas (5-7). Thirty-three cases of locally acquired murine typhus in Los Angeles County have been

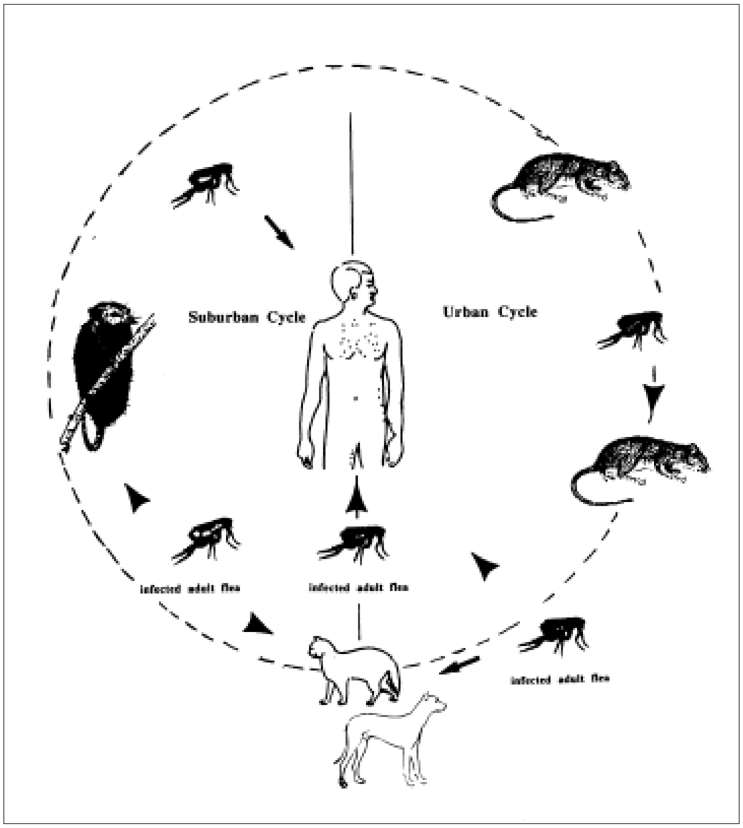

Figure 1. Urban and suburban life cycles of *Rickettsia* and mammalian hosts.

associated with seropositive domestic cats and opossums (7). More than 16 (40%) of 38 opossums and nine (90%) of 10 domestic cats collected from the case areas were seropositive for *R. typhi* antibodies. No seropositive cats (n=21) or opossums (n=36) were found in the control areas. Although flea infection was not investigated, opossums were the most heavily infested with the cat flea, *Ctencephalides felis* (104.7/animal). *C. felis* was also the most prevalent flea species (97%) collected from opossums, cats, and dogs (but not rats) in southern Texas. Surveys in other areas of the country (7,8) had similar results, which further minimizes the role of rat fleas in the maintenance of endemic typhus within the United States. The maintenance of *R. typhi* in the cat flea/opossum cycle (Figure 1) is, therefore, of potential public health importance since *C. felis* is a widespread pest that avidly bites humans (1,3,8-11).

During the past 3 years, we have investigated a new typhuslike rickettsia (initially designated as ELB and later named *R. felis*) identified in cat fleas and opossums from California and Texas murine typhus foci (8-12). Both *R. typhi* and *R. felis* were found in fleas and in opossum tissues (8-11). Additionally, a retrospective investigation of five murine typhus patients from Texas subsequently demonstrated that four patients were infected with *R. typhi* and a fifth had been infected with *R. felis* (8). Observation of human infection by *R. felis* underscores the usefulness of molecular techniques for diagnosing closely related rickettsial species. Furthermore, this documented human infection with *R. felis* and its presence in opossums and their fleas and possibly other wildlife associated with human habitation have raised concerns about the extent of *R. felis* spillover into human populations.

### The Role of Household Pets and Peridomestic Animals and Their Fleas in the Maintenance of *R. typhi* and *R. felis*

Murine typhus field surveys in southern California indicated the presence of TG antibodies in opossums, striped skunks, rats, and resident cats but not in 11 other species of native mammals (5,7). The seropositive animals were associated with human cases and were heavily infested with *C. felis* fleas. Furthermore, the isolation of *R. typhi* from opossums and their fleas led these investigators to regard opossums as of epidemiologic significance. The presence of *R. felis* in cat fleas collected from opossums within the Los Angeles murine typhus focus stimulated further interest (8,10). To understand the potential role of the *R. felis* and nonrat hosts in the biology of endemic typhus, we examined the samples collected in southern Texas, a region accounting for approximately one-third of the current reported murine typhus cases in the United States. Restriction digests of polymerase chain reaction (PCR) products from 399 cat fleas collected from nine opossums had an infection rate of 3.8% for *R. felis* and 0.8% for *R. typhi*. Three of nine tested opossums were infected with *R. felis* (8,10). No *R. typhi*-infected rats (*R. norvegicus*) or rat fleas (*X. cheopis*) were found in surveyed samples. The persistence of murine typhus in both geographic foci appears to be better accounted for by infected cat fleas, opossums, and other nonrat hosts found near human populations (Figure 1, Table 1). The presence of both *R. typhi* and *R. felis* by PCR/restriction fragment length polymorphism (RFLP) in opossums from murine typhus foci in southern California and Texas thus confirms the possible role of this marsupial in the maintenance of murine typhus infection. However, our attempt to isolate either *R. felis* or *R. typhi* from PCR-positive opossum blood or spleen collected in North Carolina, Texas, and California proved unsuccessful. Although isolating rickettsiae from blood samples has proved difficult, nevertheless, our data, as well as published information, support the role of opossums in maintaining these rickettsiae and infecting fleas.

We will now focus on the role of household pets, primarily cats, in the transmission of *R. typhi*/*R. felis*. Experimental infection of cats with *R. typhi* produces a subclinical infection with rapid recovery and seroconversion (1). Cats that were used to maintain commercial cat flea colonies displayed high antibody titers to TG rickettsiae. In addition, the report by Sorvillo et al. (7) that 90% of resident cats had demonstrable anti-TG immunofluorescence antibody assay (IFA) titers prompted us to extend our seroprevalence studies to household pets to determine the role of resident and feral cats in the urban and suburban foci of murine typhus. We accomplished this through collaboration with several veterinary schools and private practice veterinarians. For the northeast U.S. cohort, 143 cat serum samples were assayed. The sources of the samples were private practice veterinarians in the northeast United States (Michigan, New York, Ohio, Pennsylvania, Tennessee, Texas, Virginia, Wisconsin),

Table 1. Results of PCR/RFLP testing of fleas for typhus group rickettsiae

| Species | Host/Location (Date) | *Rickettsia typhi*/ *R. felis*[a] |
|---|---|---|
| *Ctenocephalides felis* | Opossum/Corpus Christi,TX(1991) | *R. felis* |
| *C. felis* | Opossum/Corpus Christi,TX(1991) | *R. typhi* |
| *C. felis* | Opossum/Los Angeles, CA(1991) | *R. felis* |
| *C. felis* | Opossum/Los Angeles, CA(1991) | *R. typhi* |
| *Polygenis gwyni* | Opossum/Statesboro, GA (1994) | -/- |
| *P. gwyni* | Opossum/Statesboro, GA (1996) | -/- |
| *Pulex simulans* | Opossum/Statesboro, GA (1993) | -/- |
| *Pulex irritans* | Dog/Statesboro, GA (1996) | *R. felis* |
| *C. felis* | Opossum/Nashville, TN (1987) | *R. felis* |
| *C. felis* | Opossum/Statesboro, GA (1993) | *R. felis* |
| *C. felis* | Opossum/Statesboro, GA (1994) | *R. felis* |
| *C. felis* | Opossum/Statesboro, GA (1996) | *R. felis* |
| *C. felis* | Dog/Statesboro, GA (1996) | *R. felis* |
| *C. felis* | Bobcat/Statesboro, GA (1994) | *R. felis* |

[a]The procedure for detecting *R. typhi* and *R. felis* in fleas followed described protocols (6,11).

who had submitted them to the Cornell University College of Veterinary Medicine, Clinical Microbiology laboratory, for testing for common feline pathogens (e.g., feline infectious peritonitis virus and feline leukemia virus). For the North Carolina cohort, 513 serum samples were tested. These were banked samples, originally collected from native cats, for use in a study of *B. henselae* seroprevalence (E.B. Breitschwerdt et al., unpub. data). IFA was used to screen cat sera for reactivity against *R. typhi* (Wilmington strain) at a 1/64 dilution. Because of the serologic cross-reactivity between *R. typhi* and *R. felis*, we have performed the definitive epitope blocking enzyme-linked immunosorbent assay (DEB-ELISA) using monoclonal anti-*R. typhi* 120 kDa surface protein antigen to rule out seropositivity to *R. typhi*. DEB-ELISA was performed on samples with an IFA titer of 1/64 (n=31 for northeast U.S. cats, n=49 for North Carolina cats) (13). For the northeast U.S. cohort, 143 samples were tested by IFA and 31 (21%) were seropositive. When these 31 positive samples were subjected to DEB-ELISA, 8 (25%) were positive, i.e., reactivity was not directed against *R. typhi*. Of 513 North Carolina cohort serum samples tested by IFA, 93 (18.1%) were seropositive at 1:64, and 49 were immunoreactive against an organism(s) other than *R. typhi*. Lack of *R. felis* monoclonal antibodies limited the usefulness of the DEB-ELISA in determining the source of infection in cats with high antibody titer against TG rickettsiae. Similar problems have been encountered in attempting to serologically identify the rickettsial species contributing to an unexplained febrile illness of dogs in the southern United States (14). Because of the antigenic cross-reactivity among the TG rickettsiae (*R. prowazekii, R. typhi*, and *R. canada*), and between the TG rickettsiae, *R. felis*, and *R. bellii*, serologic tests should be complemented by PCR/RFLP and/or direct isolation of rickettsiae by tissue culture.

A combination of IFA and DEB-ELISA used in our serosurvey represents the first attempt to define seroreactivity to TG rickettsiae among resident and feral cats. The positivity rate for these samples was unexpectedly high (18% to 21%), even though the sources of the samples were sick, febrile cats. The public health implications of these data are unclear. It is estimated that approximately one third of U.S. households have a pet cat; this translates into 57 million animals. If we conservatively estimate that only 5% of these animals have a patent rickettsial infection, 285,000 cats are possibly infected with a life-threatening zoonosis. Despite the serologic evidence presented above, the question of whether cats can serve as a reservoir host for *R. typhi* and *R. felis* and a source of infection for fleas remains to be elucidated.

### *R. typhi* and *R. felis* Infection in Fleas

Cat fleas from eight commercial colonies in various regions of the United States were infected with *R. felis* (11). The infection rates as determined by selective PCR amplification and subsequent restriction digest analysis and Southern hybridization of PCR products were 43% to 93%. These flea colonies were initiated either with fleas from one supplier, in which *R. felis* was first identified (15), or with fleas from stray cats and dogs (11). In light of the latter study (11), we recently received alcohol-preserved flea samples from L. Durden (Georgia Southern University), collected from opossums, dogs, and a bobcat in Statesboro, Georgia, and Nashville, Tennessee, between 1986 and 1996. After PCR/RFLP and partial sequencing of selected PCR products, we found that more than 50% of the cat fleas obtained from the vertebrate hosts were infected with *R. felis*. However, none of the other flea species collected from the same hosts were infected (Table 1). Although *R. felis* has been detected in cat fleas from nine states (California, Florida, Georgia, Louisiana, New York, North Carolina, Oklahoma, Tennessee, and Texas), it is probably more widespread than current data indicate. These findings demonstrate the wide distribution of *R. felis* in suburban habitats where opossums enter human habitations and share fleas with domestic dogs and cats.

Experimental infections of various laboratory colonies of fleas, e.g., *X. cheopis*, *Leptopsylla segnis*, and *C. felis*, have demonstrated a similarity in the acquisition, propagation, dissemination, and transmission of *R. typhi* (2,16). Infection in the flea is initiated when the rickettsiae, ingested in a bloodmeal, enter the midgut epithelial cells. Three to four days after infection, rickettsiae can be detected in only a small group of midgut epithelial cells (Figure 2A). Over the next 3 to 5 days, rickettsial numbers increase exponentially and spread from the initial sites of infection to the entire midgut epithelial linings. Ten days must elapse before the infected fleas can transmit *R. typhi* to susceptible hosts through infectious feces. The dynamics of *R. felis* infection have not been studied in detail experimentally, but earlier electron microscopy using infected fleas demonstrates the presence of this rickettsia in gut epithelial linings, tracheal matrix, muscle, ovaries, and epithelial sheath of the testes (15). The high rates of infection in laboratory colonies of fleas and the presence of *R. felis* in their eggs and newly emerged nonblood-fed specimens indicate that the maintenance of this rickettsiosis occurs by transovarial transmission. However, fleas may acquire both *R. typhi* and *R. felis* from rickettsemic hosts and then pass on the infection to their progeny by transovarial transmission. Neither *R. typhi* nor *R. felis* infection is lethal to fleas. There is no evidence that massive infection of flea midgut (Figure 2B) affects the feeding behavior and survival of the infected fleas. In contrast, *X. cheopis* fleas infected with *Y. pestis* starve to death while the human body lice infected with *R. prowazekii* die of the infection within 2 weeks (17). Neither *Y. pestis* nor *R. prowazekii* is maintained transovarially, and in contrast to rickettsia-infected fleas, persistence of these organisms requires constant host turnover to allow the infection cycle to perpetuate in nature. The rickettsial relationship with their arthropod hosts is considered symbiotic, yet in other instances, they act as true parasites; e.g., members of the *Rickettsia* and *Wolbachia* alter reproduction and manipulate cellular processes in their hosts (18).

Calculations based on the quantity of rickettsiae in the blood of a rickettsemic rat and the minute volume of blood ingested by fleas indicate that the invasion of flea midgut epithelium by *R. typhi* is extremely efficient, requiring only a few rickettsial organisms to result in infection (19). During the rickettsemic period in the

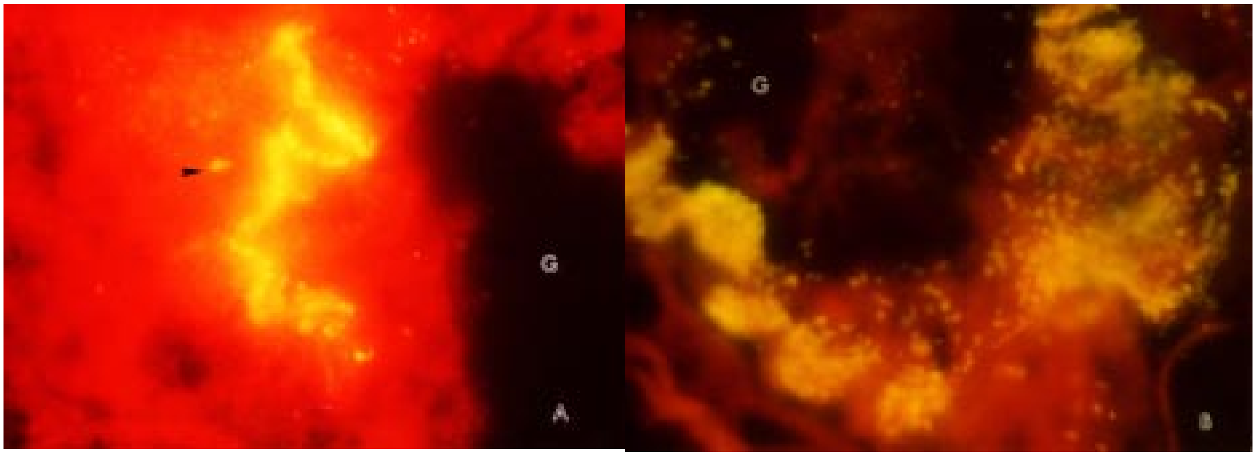

Figure 2. Direct fluorescent staining of the frozen sections of midguts of *X. cheopis* fleas showing *R. typhi*-infected epithelial cells at 3 (A) and 10 days (B) postinfectious feeding. Fleas were embedded individually in OCT compound (Miles Laboratories, Naperville, IL), sectioned (4-6 m)(16), and stained with fluorescein isothiocyanate (FITC)-labeled guinea pig anti-*R. typhi* IgG. G: gut lumen.

vertebrate host, *R. typhi* is found only in the cellular portion of the blood, particularly leukocytes. These blood cells are destroyed very rapidly (ca. 6 hours) within the flea gut (19). Experimental studies demonstrate that the rickettsial infection of flea midgut epithelium is facilitated by the flea's digestive processes. Presumably, the rapid liquefaction of the blood liberates intracellular rickettsiae from within infected blood cells. Since no peritrophic membrane is formed around the bloodmeal, there is no physical barrier to prevent freed rickettsiae from contacting and entering flea midgut epithelium. Therefore, the rapid breakdown in the cellular integrity of blood components within the flea gut lumen is probably an essential feature of the rickettsial infection of fleas. However, the biologic aspects of rickettsia-vector interactions has lagged behind other vector-borne disease studies, and only a few of the underlying phenomena involved in rickettsia-vector interactions have been partially elucidated.

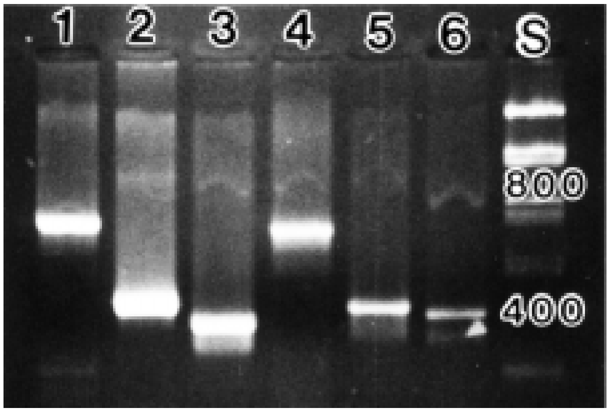

Figure 3. Amplicons for various flea-borne bacterial pathogens. Lane 1: *R. typhi* 16SrRNA (600bp); Lane 2: *R. felis* 17 kDa (434bp); Lane 3: *R. typhi* citrate synthase(384bp); Lane 4: *R. typhi* 120 kDa (612bp); Lane 5: *B. henselae* 60 kDa (414bp); Lane 6: flea 12SrRNA (414bp); and Lane S: DNA standards, with 400 and 800bp sizes indicated.

### Antigenic Characterization and Phylogeny of *R. felis*

The isolation of *R. felis* from cat flea homogenates after sequential passage from infected rat spleens through embryonated chicken eggs (20) allowed its further characterization. The identity of *R. felis* in tissue samples and fleas was achieved by a combination of PCR/RFLP (with primers based on the 17 and 120 kDa antigens, citrate synthase, and 16S rRNA gene sequences) (Figure 3), the lack of rOmpA, and seroreactivity with monospecific sera and monoclonal antibodies (Table 2,3). Furthermore, *R. felis* grown in culture more strongly resembles TG than spotted fever group rickettsiae in its morphology by light microscopy, growth pattern in Vero cells, and delayed formation of small plaques (20). Immunologic characterization of *R. felis* by a battery of monoclonal and polyclonal antibodies showed various degrees of reactivity with TG group and in particular *R. typhi* (Table 2). The T65-1 monoclonal antibody that recognizes 120 kDa surface protein of *R. typhi*, but not *R. prowazekii* or *R. canada*, reacts weakly with *R. felis*. Spotted fever group polyclonal or monoclonal antibodies also exhibited lower reactivity with *R. felis*. The SDS-PAGE profile obtained for *R. felis* here clearly resembled that of *R. typhi* and *R. akari*, lacking the prominent larger rOmpA protein found in spotted fever group rickettsiae and *R. canada* (Figure 4; 21). Immunoblot studies (20) using anti-*R. felis* polyclonal antibodies further demonstrate a strong cross-reactivity with *R. typhi* lipopolysaccharides but not spotted fever group rickettsiae.

The initial *R. felis* characterization of the sequence of a portion of the 17 kDa gene and its reactivity with anti-*R. typhi* polyclonal and monoclonal antibodies suggested resemblance to typhus rickettsiae (Table 3; 20). However, its 16S rRNA sequence more closely resembled those of *R. akari* and *R. australis*, a distinct clade of the spotted fever group (22,23). PCR amplification with rOmpA primers Rr.190.70p and Rr.190.602n

Table 2. Reactivity of *Rickettsia felis*, *R. typhi*, and *R. akari* with rat and mouse typing sera and species-specific monoclonal antibodies

| | | Reciprocal IFA titer | | |
|---|---|---|---|---|
| MAbs/Antisera | Antigen | *R. felis* | *R. typhi* | *R. akari* |
| T65-1 (IgG2a)[a] | 120kDa | 64 | 8,192 | 16 |
| 77-244-20 b | 120-135kDa | 16 | 16 | 4,096 |
| 7911-A2-H9 (IgG2a)[c] | 190kDa | 16 | 16 | 16 |
| 1F3-G2 (IgG2a)[d] | 120-135kDa | 64 | 128 | 16 |
| Rat anti-*R. felis* | | 4,096 | 2,048 | 256 |
| Rat anti-*R.typhi* | | 1,024 | 8,192 | 16 |
| Mouse anti-*R. akari* | | 512 | 16 | 4,096 |

[a]MAb against *R. typhi* 120kDa (also referred to rOmpB).
[b]MAb against *R. akari*.
[c]MAb against *R. rickettsii* 190kDa (= rOmpA).
[d]MAb against *R. rickettsii* 120-135kDa (= rOmpB).

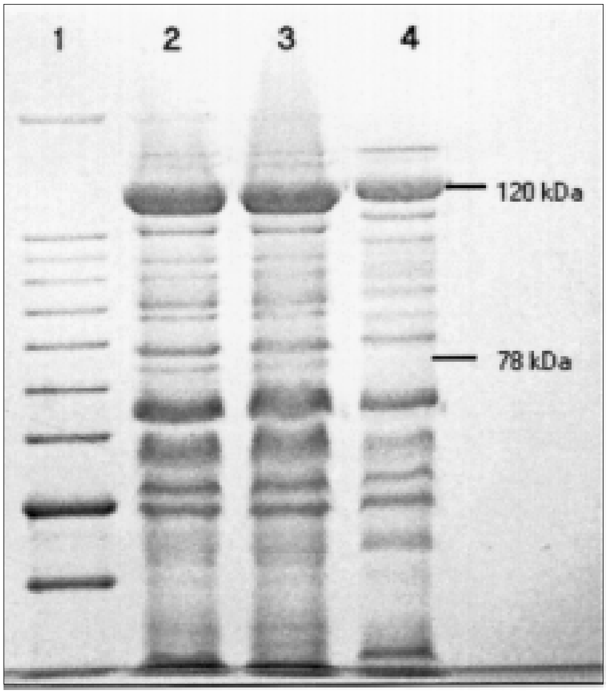

Figure 4. Coomassie blue stained polypeptide profiles of the *R. felis*, *R. typhi*, and *R. akari* plaque-purified seeds separated by SDS-PAGE (7.5%). Lane 1: 10kDa molecular mass marker, with 78 and 120kDa sizes indicated; Lane 2: *R. felis*; Lane 3: *R. typhi* (Wilmington); and Lane 4: *R. akari* (Kaplan).

was unsuccessful with *R. felis* (21). This result is perhaps not surprising since these primers also do not amplify DNA from typhus rickettsiae, *R. akari*, or *R. australis* (22,23). Recently, Andersson et al (24) reported the presence of rOmpA gene sequences in *R. prowazekii* genome, even though there is no evidence that the 190kDa protein is expressed in TG rickettsiae.

For phylogenetic studies, nucleotide sequences of all known rickettsial Citrate synthases, 17kDa surface antigen genes, and 16S r RNA sequences were obtained from Genbank and initially aligned by PILEUP (Genetics Computer Group, Madison WI) according to the progressive alignment algorithm of Feng and Doolittle (25). Nonoverlapping sequences at the trailing ends of the resulting multiple sequence file were removed and the sequences were realigned by PILEUP to give the final multiple sequence file. A distance matrix for all sequences was calculated by DNADIST, with differences between transition and transversion rates corrected according to Kimura's method (26). Evolutionary trees were computed by DNAPARS using parsimony, with the number of changes of base needed on a given tree calculated according to Fitch (27). Bootstrap analysis was performed on a 100x resampled set using SEQBOOT, with a 100x randomized input order, and the consensus tree topology was calculated by CONSENSE. The resulting cladograms for 16S rRNA and citrate synthase positioned *R. felis* within the same clade as *R. akari*, *R. australis*, and *R. helvetica*, and identified it as belonging to the spotted fever group of rickettsiae (22,23). The consensus tree for 16S rRNA is illustrated in Figure 5. The branching order within the clade comprising *R. felis*, *R. helvetica*, *R. australis*, and *R. akari* altered between different parsimony trees; however, the overall association was relatively stable. The sequence information from 17 kDa protein antigen, rOmpA, 16S rRNA, and citrate synthase genes places *R. felis* intermediate to the typhus and spotted fever group rickettsiae. The typhus and spotted fever group dichotomy, however, does not adequately reflect the evolutionary history of *R. akari*, *R. felis*, or *R. australis*.

### Future Perspectives of Typhus Infections

Traditionally TG rickettsiae have been defined by antigenic characteristics of their

Table 3. Comparison of *Rickettsia felis* with other vector-borne Rickettsiae

| Species | Vector | 190 kDa[a] | 120/ 135kDa[b] | 17 kDa[c] | Hemolysis/ plaque[d] | Phylogeny[e] |
|---|---|---|---|---|---|---|
| *R. prowazekii* | louse | - | + | + | +/+ | TG |
| *R. typhi* | flea | - | + | + | +/+ | TG |
| *R. canada* | tick | - | + | + | -/- | TG |
| *R. felis* | flea | - | + | + | +/+ | SFG |
| *R. akari* | mite | + | + | + | -/+ | SFG |
| *R. australis* | tick | - | + | + | -/+ | SFG |
| *R. rickettsii* | tick | + | + | + | -/+ | SFG |
| *R. conorii* | tick | + | + | + | -/+ | SFG |

[a]rOmpA (rickettsial outer membrane protein A). Although the presence of rOmpA gene sequences or gene product have not been shown in TG rickettsiae recently, DNA sequences corresponding to rOmpA were shown in the genome of *R. prowazekii* (Ref 24).
[b]Also referred to rOmpB.
[c]Also referred to as rickettsial inner membrane protein A.
[d]Hemolysis of sheep red cells/plaque formation.
[e]Typhus group/spotted fever group rickettsiae.
+ indicates expression of gene products/growth characteristic.

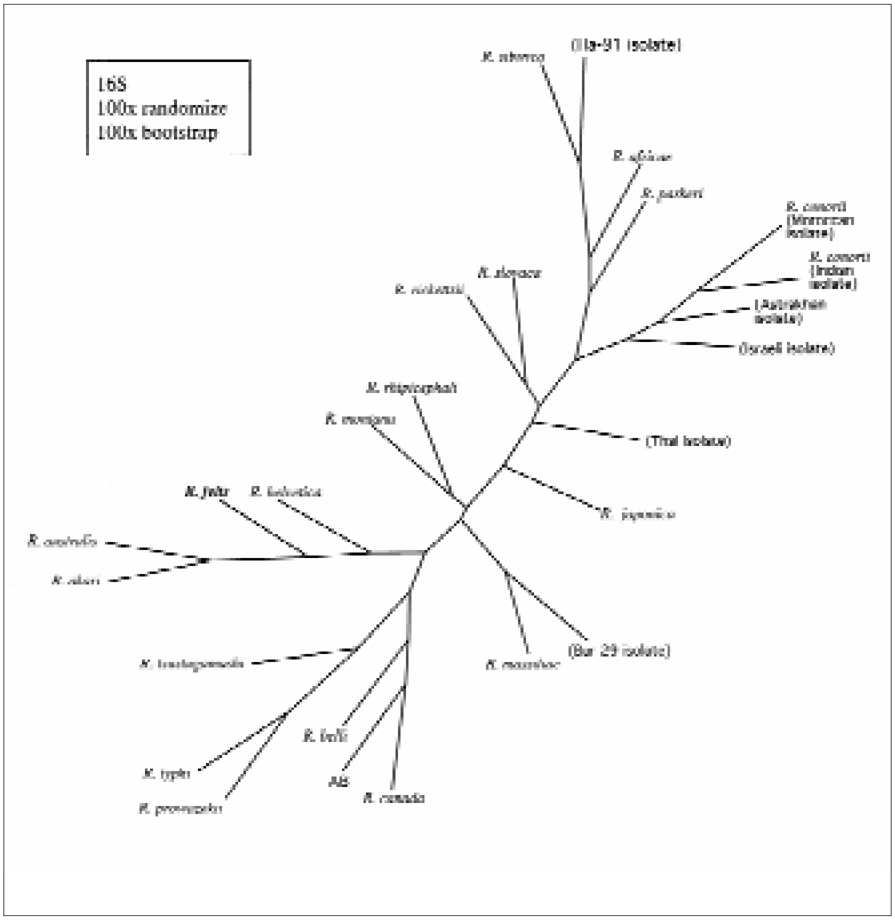

Figure 5. Consensus parsimony cladogram of rickettsial 16S ribosomal RNA sequences shows that *R. felis* (in bold) is a member of the spotted fever group.

lipopolysaccharides (4), but no fine line separates them from spotted fever group rickettsiae. Similarly the classification of rickettsiae on the basis of molecular and immunologic characterizations is also problematic because of high sequence homology and serologic crossreactivity within and between the members of TG and spotted fever group. Distinct biologic differences, however, occur between TG and SFG rickettsiae with regard to arthropod vectors, in vitro growth, antigenic repertoire, pathologic features, and clinical manifestations. Although the diagnosis can be made serologically and confirmed clinically for most of the pathogenic rickettsiae, it is unlikely to serologically distinguish *R. felis* from *R. typhi* infections. The extent of human infections with *R. felis* is unknown at this time, and the disease needs to be studied clinically. The detection and identification of *R. felis* in a single human case has been carried out by PCR/RFLP and Southern hybridization (8). The detection of both *R. typhi* and *R. felis* presents a difficult diagnostic challenge since prompt diagnosis of murine typhus or infections with *R. felis* can be established only when rickettsiae or rickettsia-PCR products from blood samples are directly isolated. DEB-ELISA using monoclonal antibodies specific to either *R. typhi* or *R. felis* would be a useful tool to differentiate between these rickettsiae and closely related species.

Reported cases of murine typhus in the United States are largely focused in central and southcentral Texas and Los Angeles and Orange Counties in California. However, murine typhus-infected rats and rat fleas are hard to document within these foci, which suggests the maintenance of *R. typhi* in the cat flea/opossum cycle. Destruction and reduction of natural habitats displace many animals and force them to move into the hospitable environments of the suburbs and cities and subsequently increase the potential for "old and new pathogens" to reemerge and generate new outbreaks. The current distribution of the opossum in more than 40 states in the United States and the invasion of urban and suburban habitats by this opportunistic marsupial have also been aided by human activities. Opossums, freeranging cats, and rats in urban and suburban habitats, where food and hospitable environments are plentiful, may live their entire lives in the same backyards. Therefore, their potential role in the murine typhus cycle as hosts to both *R. typhi*, *R. felis*, and their fleas warrants further investigation.

**Acknowledgments**

We thank Drs. G. A. Dasch, D. H. Walker, and J. A. Olson for the generous gift of monoclonal antibodies; Drs. E.

B. Breistchwerdt and Richard Jacobson for providing the cat serum samples; Dr. Colin Hunter for the phylogenetic analyses; and Mr. Kashif Firozvi for his technical assistance with the cat serology. This research was supported by a grant from the National Institutes of Health (AI 17828).

Dr. Azad is professor of microbiology and immunology, University of Maryland School of Medicine, Baltimore. His laboratory research focuses on host parasite interactions with particular interest in the molecular aspects of rickettsial pathogenesis.

### References

1. Traub R, Wisseman CL, Jr., Azad AF. The ecology of murine typhus: a critical review. Trop Dis Bull 1978;75:237-317.
2. Azad AF. Epidemiology of murine typhus. Annu Rev Entomol 1990;35:553-69.
3. Dumler JS, Taylor JP, Walker DH. Clinical and laboratory features of murine typhus in Texas, 1980 through 1987. JAMA 1991;266:1365-70.
4. Walker DH. Advances in understanding of typhus group rickettsial infections. In: Kazar J, Toman R, editors. Ricke ttsiae and rickettsial diseases. Bratislava, Slovak Republic: VEDA Press 1996;16-25.
5. Adams WH, Emmons RW, Brooks JE. The changing ecology of murine (endemic) typhus in southern California. Am J Trop Med Hyg 1970;19:311-8.
6. Williams SG, Sacci JB Jr, Schriefer ME, Anderson EM, Fujioka KK, Sorvilo FJ. Typhus and typhus-like rickettsiae associated with opossums and their fleas in Los Angeles county, California. J Clin Microbiol 1992;30:1758-62.
7. Sorvillo FJ, Gondo B, Emmons R, Ryan P, Waterman SH, Tilzer A, et al. A suburban focus of endemic typhus in Los Angeles County: association with seropositive domestic cats and opossums. Am JTrop Med Hyg 1993;48:269-73.
8. Schriefer ME, Sacci JB Jr, Dumler JS, Bullen MG, Azad AF. Identification of a novel rickettsial infection in a patient diagnosed with murine typhus. J Clin Microbiol 1994;32:949-54.
9. Azad AF, Sacci JB Jr, Nelson WM, Dasch GA, Schmidtman ET, Carl M. Genetic characterization and transovarial transmission of a novel typhus-like Rickettsia found in cat fleas. Proc Natl Acad Sci USA 1992;89:43-6.
10. Schriefer ME, Sacci JB Jr, Higgins JA, Taylor JP, Azad AF. Murine typhus: updated role of multiple urban components and a second typhus-like rickettsiae. J Med Entomol 1994;31:681-5.
11. Higgins JA, Sacci JB Jr, Schriefer ME, Endris RG, Azad AF. Molecular identification of rickettsia-like microorganisms associated with colonized cat fleas (*Ctenocephalides felis).* Insect Mol Biol 1994;3:27-33.
12. Higgins JA, Radulovic S, Schriefer ME, Azad AF. *Rickettsia felis*: a new species of pathogenic rickettsia isolated from cat fleas. J Clin Microbiol 1996;34:671-4.
13. Radulovic S, Speed R, Feng HM, Taylor C, Walker DH. EIA with species-specific monoclonal antibodies: a novel seroepidemiologic tool for determination of the etiologic agent of spotted fever rickettsiosis. J Infect Dis 1993;168:1292-5.
14. Breitschwerdt EB, Hegarty BC, Davidson MG, Szabados NS. Evaluation of the pathogenic potential of *Rickettsia canada* and *Rickettsia prowazekii* organisms in dogs. JAVMA 1995;207:58-63.
15. Adams JR, Schmidtmann ET, Azad AF. Infection of colonized cat fleas, *Ctenocephalides felis* with a rickettsia-like microorganism. Am J Trop Med Hyg 1990;43:400-9.
16. Azad AF, Traub R, Sofi M, Wisseman CL Jr. Experimental murine typhus infection in the cat flea, *Ctenocephalides felis* (Siphonaptera:Pulicidae). J Med Entomol 1984;21:675-80.
17. Azad AF. Relationship to vector biology and epidemiology of louse and flea-borne rickettsioses. In: Walker DH, editor. Biology of rickettsial diseases. Boca Raton (FL): CRC Press; 1988. p.52-62.
18. Werren JH, Zhang W, Guo W. Evolution and phylogeny of *Wolbachia*: reproductive parasites of arthropods. Proc R Soc Lond B Biol Sci 1995;B261:55-71.
19. Vaughan JA, Azad AF. Acquisition of murine typhus rickettsiae by fleas. Ann NY Acad Sci 1990;590:70-5.
20. Radulovic S, Higgins JA, Jaworski DC, Dasch GA, Azad AF. Isolation, cultivation and partial characterization of the ELB agent associated with cat fleas. Inf Immun 1995;63:4826-9.
21. Regnery RL, Spruill CL, Plikaytis BD. Genotypic identification of rickettsiae and estimation of intraspecies sequence divergence for portions of two rickettsial genes. J Bacteriol 1991;173:1576-89.
22. Roux V, Raoult D. Phylogenetic analysis of the genus Rickettsia by 16S rDNA sequencing. Res Microbiol 1995;146:385-96.
23. Stothard D, Fuerst PA. Evolutionary analysis of the spotted fever and typhus group of *Rickettsia* using 16S rRNA gene sequences. Systematic Applied Microbiology 1995;18:52-61.
24. Andersson S, Eriksson A-S, Naslund AK, Andersen MS, Kurland CG. The *Rickettsia prowazekii* genome: a random sequence analysis. Microbial and Comparative Genomics 1996;1:293-315.
25. Feng DF, Doolittle RF. Progressive sequence alignment as a prerequisite to correct phylogenetic trees. J Mol Evol 1987;25:351-60.
26. Kimura M. A simple model for estimating evolutionary rates of base substitutions through comparative studies of nucleotide sequences. J Mol Evol 1980;16:111-20.
27. Fitch WM. Toward defining the course of evolution: minimum change for a specified tree topology. Systematic Zoology 1971;20:406-616.
